# Health care utilization and cost after discharge from a mental health hospital; an RCT comparing community residential aftercare and treatment as usual

**DOI:** 10.1186/s12888-018-1941-2

**Published:** 2018-11-12

**Authors:** Eirik Roos, Ottar Bjerkeset, Aslak Steinsbekk

**Affiliations:** 1 Department of Public Health and Nursing, Norwegian University of Science and Technology, 7491 Trondheim, Norway; 2 Health and Welfare, Trondheim, Norway; 3 Faculty of Nursing and Health Sciences, Nord University, Levanger, Norway; 4 Department of Mental Health, Norwegian University of Science and Technology, Trondheim, Norway

**Keywords:** Community residential aftercare, Step-down, Discharge-ready mental health patients, Severe mental illness

## Abstract

**Background:**

Community residential aftercare (step-down) services can ease the transition after a mental health hospital stay for patients with severe mental illness (SMI).

**Aims:**

To investigate use of community and specialised mental health care services and costs in patients with SMI the first 12 months after discharge from a mental health hospital (MHH), comparing community residential aftercare (CRA) and treatment as usual.

**Methods:**

An open parallel group randomised controlled trial with 41 participants. Data on use of specialist services (hospital, ambulant treatment and outpatient treatment) and community services (residential stays, home help, home care nursing, mental health consultation) were collected from specialist and community registers and health records.

**Results:**

For the primary outcome, utilisation of community mental health services, the intervention group used, on average, 29% fewer hours (mean differences − 21.6 h, 95% CI -93.1 to 44.9, *p* = .096) with a cost saving of 29% (mean differences − 1845 EUR, 95% CI -8267 to 4171, *p* = .102), but the estimates were imprecise. For the secondary outcome, the study groups had the same total number of inpatient days (66 days), but the intervention group had on average of 13.4 fewer inpatient days in the mental health hospital (95% CI -29.9 to 0.9. *p* = .008). The number of inpatient admissions (mean difference − 0.9 admissions, 95% CI -3.5 to 1.5, *p* = .224) and readmissions (− 0.8, 95% CI -2.5 to 0.9. *p* = .440) was lower in the intervention group. The intervention group had on average a total cost saving of 38.5% (mean differences − 23,071 EUR, 95% CI -45,450 to 3027. *p* = .057). A post hoc multivariable regression analysis controlling for baseline characteristics gave a reduction in total cost in favour of the intervention group of − 19,781 EUR (95% CI -44,072 to 4509, p=,107).

**Conclusion:**

In this study, it was not possible to draw a definite conclusion about the effect, due to the small sample and imprecision of the estimates. The direction of the results and size of the point estimate, in addition to findings in other studies, indicates that transferring patients ready for discharge from mental hospital to community residential aftercare can have the potential to reduce total consumption of health services and costs without increased hospital admissions.

**Trial registration:**

Registered in clinicaltrials.gov (NCT01719354)

## Background

Most psychiatric inpatients can be discharged without comprehensive follow-up, yet patients with severe mental illness (SMI) often need long-term aftercare [1]. This is a particularly vulnerable group, as patients with SMI have a 10–25 year shorter life expectancy than the general population [2]. Furthermore, a Danish population-based cohort study found increased risk of hospitalisations and rehospitalisation within 30 days for patients with SMI compared with the general population [3].

The duration of hospital stays is a major driver for health costs [4] and most Western countries have shifted more mental health care towards community-based settings [5]. However, it is a challenge to provide timely community services for patients who are ready for discharge from mental health hospitals. A study in the UK in 2005 found that the proportion of discharges classified as “delayed” varied from 4 to 16% of all hospital beds [6]. A study from Norway in 2013 found that 7% of all patients in mental health hospitals were ready for discharge, but were still waiting for municipal services to take over, mainly to provide sheltered housing [7]. A review of 35 studies, mostly from general hospitals, on delayed discharge [8] found that the average cost of one extra day per patient was between £200 and £565.

Early psychiatric readmission serves as a negative quality of care indicator in the mental health services [9, 10]. Some studies report that short inpatient treatment stays (< 28 days) increase readmission rates [11–13]. In contrast, a Cochrane review from six randomised studies did not find evidence suggesting that short-stay hospitalisation (< 28 days), compared to long stay (> 28 days), encouraged a ‘revolving door’ pattern of admission to hospital [14].

Community based residential mental health services can serve as an alternative to both inpatient admissions (step-up) and aftercare (step-down). A review from 2013 [15] evaluated such services for acute [16–18] and sub-acute admissions (step-up) [19] and concluded that these step-up residential community services offered a cost-effective alternative to hospital based inpatient services. Similarly, a few studies have evaluated community-based services in the form of residential aftercare after hospital stays (step-down) [20–23]. An RCT study on inpatient treatment for substance use disorders compared the effects of two types of community-based, residential treatment programs among justice involved persons with dual diagnosis and reported significant reductions in psychiatric severity for those assigned to residential conditions [23]. An observational study found that a staffed residential step-down facility with a comprehensive program improved symptoms and functioning for persons with psychosis or mood disorder [21].

Taken together, this indicates that patients ready for discharge could be discharged as early as possible to a community residential service, without the shorter stay leading to increased risk of readmission [14], and the costs would be reduced [8]. To make this happen for in-patients with SMI, there is a need for improved collaboration and communication between service levels [24, 25] as well as services that can receive patients who need community services after their hospital stay [20].

There is, however, still a need for studies on the effect and costs of residential aftercare services in the community. One type not previously investigated, is residential aftercare services that do not offer organised in-house activities. Offering organised in-house activities may substitute for future local activities and integration in the local community. Thus, not offering in-house activities could potentially help patients use community services more actively and promote more independent living. The reason being that the patients would have to orient themselves more towards the activities in the community during the stay [26].

The aim of this RCT study was to investigate use of community and specialist mental health care services and costs in patients with severe mental illness (SMI) the first 12 months after discharge from a mental health hospital (MHH), comparing community residential aftercare (CRA) and treatment as usual.

## Methods

This was an open parallel group randomised controlled trial including patients from January 2013 to April 2015. It was approved by the Committee for Medical and Health Research Ethics in Central Norway (2011/1770) and was registered in clinicaltrials.gov (NCT01719354).

### Change to protocol

Fewer patients than aimed for were included due to problems with recruitment (59% of calculated sample size). It was planned to collect self-reported outcome at 1, 4 and 12 months, but it proved very difficult to get the participants to complete the questionnaires even after 1 month despite several attempts. The collection of these data was therefore stopped, meaning that only outcomes on the consumption of health care services and costs as outcomes were used.

### Settings

In Norway, the health and social care services are mainly financed by and provided for in the public sector [27]. Community health and long-term care is the responsibility of the municipalities, while acute somatic and psychiatric hospitals and specialist services are run by the government. Community health and social care includes GPs, public health nurses, nursing homes, home care and mental health care (some places including residential care). Specialist health care organises acute and psychiatric specialist services into mental health hospital (MHH), community mental health centre (CMHC), mental health outpatient treatment and mental health ambulant treatment.

In central Norway, community residential aftercare units (CRA) have been established in order to improve the discharge process from hospital to independent supported living [26]. They facilitate the process of establishing community health and social services, support self-care and engagement, but do not offer organised in-house activities, to ensure community orientation and the fostering of initiatives among the patients. Both the community residential aftercare (CRA) unit and the university mental health hospital (MHH), the setting for this study, are in the City of Trondheim (190,000 inhabitants), in central Norway. The municipality of Trondheim offers a multitude of mental health services to people with mental disorders: community mental health consultation, home care nursing, home help, day centre, short-stay residential aftercare, self-referral and housing arrangement. The MHH has 81 beds, half for acute admissions and half for long-stay patients.

### Eligibility criteria

All in-patients with severe mental illness (SMI) at the MHH who were assessed as discharge ready and in need of aftercare services from the municipality after discharge were eligible for this study. However, they had to have a treatment aftercare plan initiated by the time of inclusion. Furthermore, there were no requirements regarding specific diagnostic criteria, and this group mainly concerns people with a diagnosis of schizophrenia, schizoaffective disorders, bipolar disorder, major depression or personality disorders. Furthermore, the patients had to be older than 18 years and they had to sign the informed consent. The exclusion criteria were patients with impaired level of consciousness or acute confusion, those who were under involuntary observation or admission according the Norwegian mental health care act (those involuntary admitted were included if it had been converted to voluntary hospitalisation) and patients assessed by the hospital to be without need of community services after discharge.

### Recruitment

All patients were recruited at the MHH in both acute and long stay departments after they were declared by the hospital to be ready for discharge. Staff in the departments identified eligible patients. The doctors in the hospital were responsible for assessing whether the patients were able to understand the consequences of participating in the study. The hospital nurses were the ones mainly responsible for informing the patients orally about the study and giving them written information and the informed consent. The patients were given one day to decide on their participation and those who wanted to take part signed the consent and gave it to the staff who collected baseline data.

### Randomisation and allocation

The randomisation was done using a web-based computer program provided by a trial service at the Norwegian University of Science and Technology. The staff at the MHH conducted the randomisation after receiving the informed consents and the baseline data, and they informed the patients about the allocation.

### Intervention – The CRA

A more detailed description of the community residential aftercare unit has been published previously [26]. Briefly, the CRA was established in 2009 and has 14 rooms in total. A stay at the CRA is voluntary and the tentative length of a stay is up to 4 w, but for homeless patients the stay is longer due to the practicalities of making housing arrangements (14 homeless patients in 2016 had an average stay of 64 days) [26].

The CRA operates 24/7 and is staffed by psychiatric nurses, general nurses and nursing assistants. A general practitioner (GP) is present in the CRA one day a week and offers a consultation to all patients who have recently been admitted, and those in need of medical follow-up at the CRA.

The philosophy of the CRA involves the conscious decision not to offer any in-house activities. Instead, the patients are informed about activities in their neighbourhood and in the community. Therefore, there are no organised activities at the CRA such as meals in common, therapy options or use of exercise equipment.

The CRA staff facilitates the process of establishing community health and social services to support the transition from the hospital to independent supported living. The process is started as early as possible to establish a relationship between the patient, the responsible case handler in the municipality and the service providers offering follow-up services after discharge. During the stay, the result of the individual assessment is discussed with the patient, the case handler and it is communicated to the community Health and Welfare agency to help it to decide on the level of services provided by the municipality after discharge. Before discharge from the CRA, patients receive information about the possibility of later self-referral to a short (maximum of three days) inpatient stay at the CRA.

### Control – Treatment as usual (TAU)

The TAU discharge process in the MHH for discharge ready patients in need of community follow-up typically includes one of the following: (1) The staff in the hospital contact the Health and Welfare agency in the municipality to clarify which type of follow-up services are needed from the municipality, including housing. This is settled before discharge to the home. (2) The staff in the hospital refers the patient to a community mental health centre (CMHC), which is part of the specialist services, where they continue the treatment plan initiated by the MHH before the CMHC contacts the municipality to make plans before discharge to home.

#### Measures

To document the implementation of the intervention, the following data were collected: (1) days in the MHH before randomisation (expected to be equal between the groups). (2) Days from randomisation to discharge (expected to be shorter in the intervention group). (3) Where they were discharged immediately after the index stay in the MHH (only the intervention group should be discharged to the CRA). (4) The length of stay at an inpatient unit or residential unit immediately after the index stay (expected to be longer in the intervention group).

### Primary outcome

The primary outcome was total hours of community health services and costs for these services during a 12-month period. This included total number of hours with home help (cleaning, shopping etc.), home care nursing and community mental health consultation. The reason for having this as the primary outcome was that it was expected based on experience that patients discharged to the CRA was assessed to need less community services compared to the assessment made based on observation in a hospital setting.

### Secondary outcome

The secondary outcomes were number of and cost for the total inpatient days in the MHH, CMHC and CRA, total hours with outpatient treatment including ambulant treatment and the total number of admissions and readmissions from baseline to 12 months after inclusion. Readmission was defined as acute, unplanned admissions to the MHH, CMHC or the CRA within 30 days after last discharge. As a summary measure for the secondary outcomes, total cost of all services was used.

### Data collection

All data were provided by the staff in the community health and social care and specialist health care services. They collected the data from registries with data on contacts with the services (“consultations”) which are registered with a very high grade of accuracy as it is both demanded by law to be registered and in the interest of the services to do so as it is connected to the use of resources and thus financing. In addition, data on patient characteristics was collected at baseline.

### Calculation of cost

The cost of the different services was provided by employees in the administration of the municipality of Trondheim and the university hospital, using the cost from 2015 (Table 1). These figures included the total staff costs, rent and operating expenditures.

**Fig. 1:**
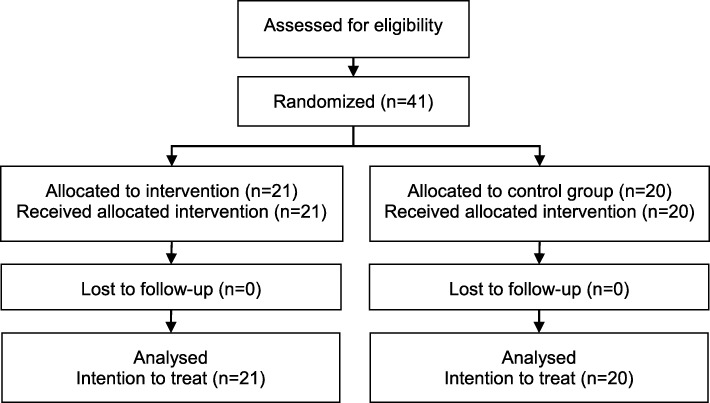
Flow chart

**Table 1:** Cost in 2015 per inpatient day and per hour for various mental health services, with the sector responsible for financing

Place	Cost	Financed by university hospital	Financed by municipality
Cost per inpatient day (24 h)			
Mental health hospital	1065 EUR	X	
Community mental health centre	619 EUR	X	
Community residential aftercare^a^	270 EUR		X
Cost per hour:			
Outpatient treatment at hospital	292 EUR	X	
Ambulant treatment^b^	181 EUR	X	X
Home help	84 EUR		X
Home care nursing	84 EUR		X
Community mental health consultation	90 EUR		X

The exchange rates were €100 = 948.50 NOK, rate at the Norges Bank on 05.07. 2017 using the mid-price (the midpoint between the buying and selling price)

^a^For the community residential aftercare, all operating cost (staff cost, and all expenditures) in 2015 was 805,738 EUR excluding capital cost. The operating cost was divided by 14 beds and 365 days and gave a cost of 184 EUR per inpatient day. The capital costs used was the mean of all nursing homes and residential aftercare units in the municipality (86 EUR)

^b^For the ambulant treatment, the cost was recalculated, as the cost provided by the administration (1168 EUR per hour) seemed too high. This was due to the hour cost being higher than the cost of one day in the hospital, and those providing the cost figures could not specify this figure. The recalculation was based on the yearly budget in 2015 of 1,062,309 EUR. It was assumed that the 10-full time equivalent employees had face-to-face time contact with the services user in 1/3 (due to travel, sometimes more than one employee visiting, administrative work etc.) of their work-time. One full time equivalent equals 1750 h per year (37.5 h per week). This gives some 5800 h of face-to-face services to the approximately 100 users receiving this service. This corresponds well with the number of hours of ambulant treatment observed in the trial among those receiving such services (median 53 h)

Cost is in EUR

#### Sample size

As there were no publications on which to base the power calculation, it was based on historical data (one month in 2012) from the municipal health registers for 14 patients who had stayed at the CRA and 13 who had been discharged directly from MHH. The mean number of hours of community care services per week was 3.7 (SD 3.5) for CRA patients and 20.91 (SD 40.4) for MHH patients. Mean daily function (ADL) score for CRA patients on a 1–5 scale was 1.58 (SD 0.37) and it was 1.94 (SD 0.65) for MHH patients.

Including 35 patients in each group, using a two-tailed *t*-test with a 5% statistical significance level and power of 80% would detect these differences. The aim was to include a total sample of 140 to allow for an expected high dropout and withdrawal rate.

#### Blinding

There was no blinding of the patients or staff due to the nature of the intervention. The persons extracting the data from the registers were not aware of the allocation. The outcome data only included data registered as part of the patients’ regular care and, therefore, could not be influenced by the study staff.

#### Statistical methods

The comparison between the groups was based on the intention to treat principle, where the participants were analysed according to the group they were randomised to. No per protocol test was planned or done. There were complete data on the use of all the outcomes for all participants, meaning that no measures had to be taken regarding missing. Due to the outcome data having a strong non-normal distribution and outliers, and the small sample size (*n* = 41), the comparison of the continuous variables was analysed with the non-parametric Mann-Whitney U-test [28]. The categorical data were calculated using Pearson chi square or Fisher exact test.

The outcomes in the groups is presented with both median and mean values and mean difference with 95% confidence interval (95% CI) which were calculated using t-tests with bootstrapping for the continuous data. Thus, the 95% CI (from the parametric test) does not correspond to the *p*-values reported (from non-parametric test). For the categorical data, the difference is presented in percentage points.

There were some differences between the characteristics of the groups at baseline. Therefore, a post hoc analysis was done using linear regression analysis with total cost as dependent variable and baseline variables as independent variables. Total cost was chosen as it best captures the overall picture of the participants’ health care use. Due to the small sample, and the rule of thumb of having at least 10 observation for every variable included in a regression analysis [29], only the baseline variables with more than 20%-point differences between the groups (Table 2, Homeless, Diagnosis, Employment status, Living alone) were included in the model as independent variables in addition to group allocation.

All analyses were done with SPSS 24 for Windows (IBM Corp. Armonk, NY).

## Results

### Participants flow

The total number of participants assessed for eligibility was not registered. However, in the weekly meetings between the researcher and the contact nurses (one nurse from each department in the MHH), the nurses reported that almost all participants who were introduced to the study, said that they would participate. Forty-one participants met the inclusion criteria and were randomised Fig. 1.

### Baseline data

There were some differences between the groups on some variables at baseline. There were more patients living alone, being homeless and unemployed in the intervention group, with one patient with a F6 diagnosis (personality disorder). In the control group, (Table 2) more patients were involuntary admitted and had a F6 diagnosis.

**Table 2:** Demographic variables and diagnosis for patients at baseline

	All (*n* = 41)	Intervention (*N* = 21)	Control (*N* = 20)	Difference in % points
Age, mean (SD)	42.9 (14.7)	42.2 (14.9)	43.8 (14.8)	0
Female	21 (51%)	9 (43%)	12 (60%)	− 17
Living alone	29 (71%)	17 (81%)	12 (60%)	21
Homeless	15 (37%)	12 (57%)	3 (15%)	42
Sheltered housing	0	0	0	0
Involuntary admitted	8 (20%)	3 (14%)	5 (25%)	− 11
Employment status				
Full-time employment	2 (5%)	1 (5%)	1 (6%)	−1
Part-time employment	2 (5%)	2 (10%)	0 (0%)	10
Unemployment	10 (24%)	8 (40%)	2 (13%)	27
Disability pension	23 (56%)	10 (48%)	13 (65%)	− 17
Student	1 (2%)	0 (0%)	1 (6%)	− 6
Highest level of education				
Compulsory school	11(31%)	7 (37%)	4 (23%)	− 14
Middle level education	20 (55%)	9 (47%)	11 (65%)	− 18
Higher education	5 (14%)	3 (16%)	2 (12%)	4
Main Diagnosis (ICD- 10 code)				
Mental and behavioral disorders (F1)	4 (10%)	2 (10%)	2 (10%)	0
Schizophrenia, schizotypal, delusional disorders (F2)	10 (24%)	5 (24%)	5 (24%)	0
Mood (affective) disorders (F3) and anxiety disorders (F4)	17 (41%)	12 (57%)	5 (24%)	33
Behavioral and personality disorders (F6)	5 (12%)	1 (5%)	4 (20%)	−15
Observation for suspected mental and behaviour disorders (Z03.2)	5 (12%)	1 (5%)	4 (20%)	−15

N varies due to missing: Employment (control: three missing). Education (intervention: two missing. Control: three missing)

Numbers are N (%) except for age which is mean (SD)

### Implementation of the intervention

The intervention was implemented as planned, with changes in the observed variables in the direction expected (Table 3). All patients in the intervention group were discharged to the CRA. The difference in mean length of mental hospital inpatient stay (LOS) from randomisation to discharge was 6.3 days (3.8 days in the intervention group and 10.1 days in the control group, *p* = .023).

**Table 3:** Implementation of the intervention

Variable	All (*n* = 41)	Intervention (*N* = 21)	Control (*N* = 20)	*P*-value
Discharged to	*N* (%)	*N* (%)	*N* (%)	
Home	11(27%)	0 (0%)	11 (55%)	
CMHC	9 (22%)	0 (0%)	9 (45%)	
CRA	21 (51%)	21 (100%)	0 (0%)	
Number of hospital inpatient days from index admission to discharge from MHH				
Mean (SD)	18.3 (26.9)	20.4 (30.9)	16.1 (22.5)	
Median (IQR), range	11 (5–16), 113	9 (4.3–17.5), 112.5	12.5 (6.5–16), 107	.531
Number of hospital inpatient days from index admission to date of randomisation (baseline)				
Mean (SD)	11.4 (19.9)	16,6 (26.9)	6.0 (4.1)	
Median (IQR), range	6 (3–10.5), 90	6 (2.5–13), 90	6 (3–8.5), 14	.495
Number of hospital inpatient days from date of randomisation (baseline) to discharge date				
Mean (SD)	6.9 (15.4)	3.8 (5.8)	10.1 (21.1)	
Median (IQR), range	3 (1–7), 97.5	1 (1–4), 23.5	4.5 (1.3–10), 97.0	.023
Length of stay at an institution immediately after discharge from the mental health hospital				
CMHC				
Mean (SD)	5.9 (12.8)	0 (0)	12 (16.4)	
Median (IQR), range	0 (0–0), 55	0 (0–0), 0	0 (0–23), 55	
CRA				
Mean (SD)	24.1 (35.6)	45.9 (37.6)	0 (0)	
Median (IQR), range	1.5 (0–44), 176	44 (28–58), 175	0 (0–0), 0	

*MHH* Mental health hospital, *CMHC* Community mental health centre, *CRA* Community residential aftercare

#### Outcomes

**Fig. 2:**
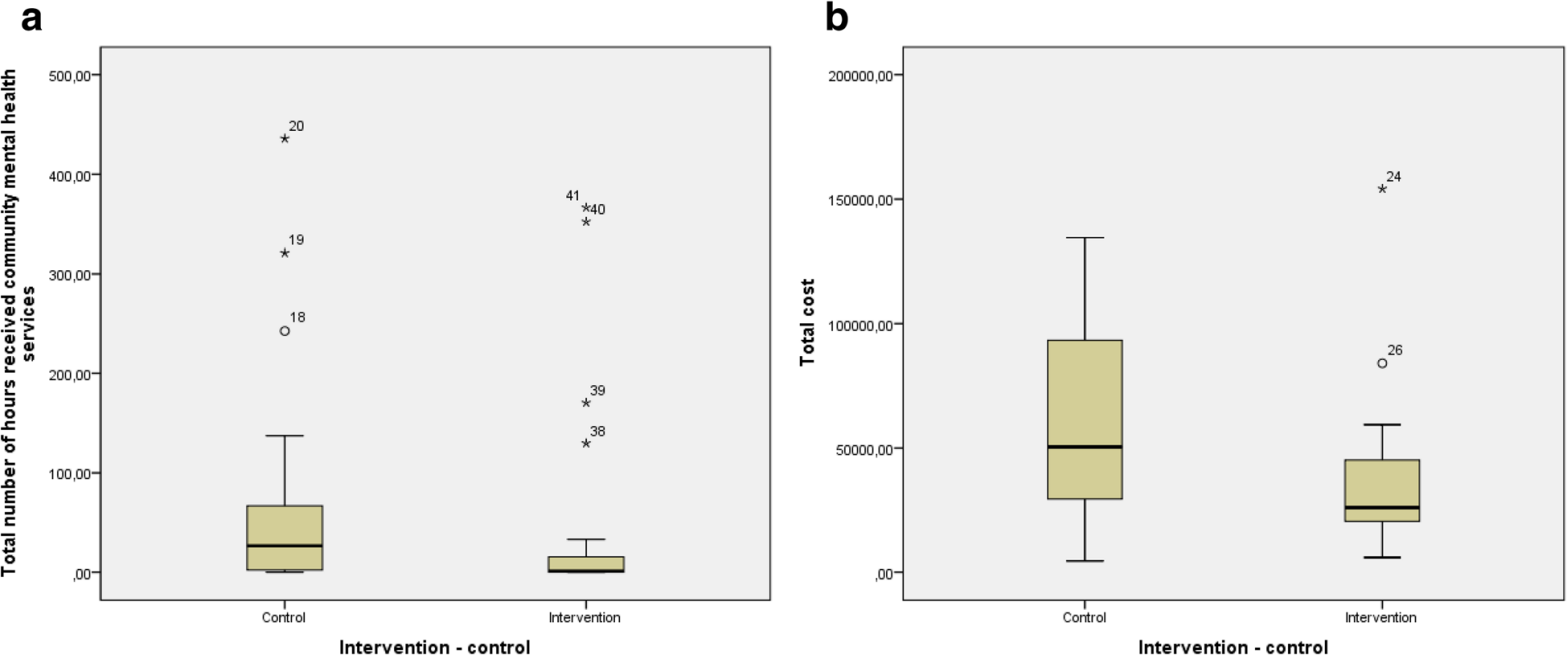
Box plot for the primary outcome total number of hours of community mental health services (**a**), and the secondary outcome total cost of specialist and community health services in euro (**b**) from baseline to twelve months

There were large variation and some outliers for most of the outcomes (Fig. 2).

### Primary outcome

Those randomised to the CRA had on average 29% fewer hours of community mental health services for 12 months but the precision of the estimate was low, i.e. wide confidence intervals (mean difference − 21.6 h, 95% CI -93.1 to 44.9, *p* = .096) (Table 4). This difference was mainly due to less use of home care nursing. The cost for the community mental health services was 29% lower with a mean difference of − 1845 EUR (95% CI -8267 to 4171, *p* = .102) with similar imprecision in the estimates.

**Table 4:** Primary outcomes: Total number of hours, number of patients and costs of community mental health services from baseline (date of randomisation) to 12 months

Variable	All (*n* = 41)		Intervention (*N* = 21)	Control (*N* = 20)	Between groups	*P*- value
Number of hours	Mean (SD)	Median (IQR), range	Mean (SD)	Mean (SD)	Mean diff (95% CI)	
Total number of hours received community mental health services	62.9 (115.1)	6.0 (0.0–54.0), 436.0	52.4 (111.4)	74 (120.8)	−21.6 (−93.1 to 44.9)	.096
Home help (cleaning, shopping etc.)	1.8 (6.6)	0 (0–0), 39.3	1.4 (3.7)	2.3 (8.8)	−0.9 (−5.5 to 2.4)	.680
Home care nursing	55.3 (106.3)	0 (0–49.4), 396.7	47.4 (111.0)	63.5 (103.4)	−16 (−85.5 to 51.2)	.023
Community mental health consultation	5.9 (20.7)	0(0–3.3), 128.6	3.6 (8.2)	8.2 (28.6)	−4.5 (− 20.9 to 4.5)	.758
Number of patients	*N* (%)		*n* (%)	*n* (%)	Difference in %- points	
Total number of patients with the listed services^a^	29 (71%)		13 (62%)	16 (80%)	− 18	.209
Home help (cleaning, shopping etc.)	5 (12%)		3 (14%)	2 (10%)	4	.679
Home care nursing	20 (49%)		6 (29%)	14 (70%)	− 41	.009
Community mental health consultation	14 (34%)		8 (38%)	6 (30%)	8	.589
Costs	Mean (SD)	Median (IQR), range	Mean (SD)	Mean (SD)	Mean diff (95% CI)	
Total cost of Community mental health services	5345 (9752.6)	546 (0–4587), 36,773	4444.7 (9399)	6290.5 (10,266.7)	− 1845 (−8267 to 4171)	.102
Home help (cleaning, shopping etc.)	153 (558)	0 (0–0), 3313	116 (310)	191 (743)	−74 (− 476 to 200)	.680
Home care nursing	4661 (8966)	0 (0–4165), 33,460	3999 (9359)	5357 (8721)	− 1357 (− 7124 to 4683)	.023
Community mental health consultation	530 (1864)	0 (0–300), 11,605	328 (742)	741 (2577)	− 413 (− 1784 to 457)	.758

^a^Each patient could receive more than one service

### Secondary outcomes

The total number of inpatients days after discharge from the initial stay to 12 months was 66 days for both groups (Table 5), but patients randomised to the CRA had 54% fewer inpatient days in the MHH (mean differences − 13.4 days, 95% CI -29.9 to 0.9, *p* = .008). About half (6.3 days, Table 3) of the difference between the groups in MHH inpatients days (13.4 days) was due to the patients in the control group being discharged later from the MHH after the initial stay. The total number of admission to any institution after the initial stay was 3.9 times in the intervention group and 4.9 times in the control group (mean difference − 0.9 times, 95% CI -3.5 to 1.5, *p* = .224).

**Table 5:** Secondary outcomes. Number of mental health inpatient days, number of admissions and number of readmission < 30 days from baseline (date of randomisation) to twelve months

Variable	All (*n* = 41)		Intervention (*N* = 21)	Control (*N* = 20)	Between groups	*P*- value
	Mean (SD)	Median (IQR), range	Mean (SD)	Mean (SD)	Mean diff (95% CI)	
Total inpatient days	66.5 (61.4)	50 (34.5–77.5), 306	66.7 (55.9)	66.4 (68.1)	0.3 (−35.8 to 40.3)	.629
MHH	17.8 (25.0)	8 (3–18), 97.5	11.2 (19.8)	24.7 (28.5)	−13.4 (−29.8 to 0.9)	.008
CMHC	17.9 (31.5)	0 (0–27.5), 120	7.9 (22.3)	28.4 (36.7)	−20.4 (−38.8 to − 2.8)	.004
CRA	29.7 (50.3)	2 (0–47.5), 255	45.9 (37.6)	12.8 (57.0)^a^	33.2 (− 0.1 to 60)	.000
CRA self-referral	1.2 (2.8)	0 (0–0), 12	1.7 (2.8)	0.6 (2.7)^b^	1.1 (− 0.6 to 2.7)	.035
Total number of admission after initial stay	4.4 (4.0)	3 (1–5), 15	3.9 (3.9)	4.9 (4.1)	−0.9 (−3.5 to 1.5)	.224
MHH	2.8 (2.9)	2 (1–3.5), 12	2.6 (2.8)	3.1 (3.0)	−0.5 (−2.3 to 1.2)	.358
CMHC	1.2 (2.5)	0 (0–2), 14	0.7 (1.7)	1.8 (3.1)	−1.0 (−2.8 to 0.3)	.016
CRA	0.5 (0.5)	1 (0–1),1	1.0 (0.0)	0.05 (0.22)^a^	0.9 (0.8 to 0.9)	0.00
CRA self-referral	0.4 (0.83)	0 (0–0), 3	0.7 (1.1)	0.05(0.22)^b^	0.6 (0.2 to 1.1)	.019
Total number of readmissions after initial stay	1.5 (2.8)	0 (0–2.5), 11	1.2 (2.4)	1.9 (3.2)	−0.8 (−2.5 to 0.9)	.440
MHH	1 (1.9)	0 (0–1), 8	0.81 (1.7)	1.2 (2.2)	−0.4 (−1.7 to 0.8)	.820
CMHC	0.5 (1.9)	0 (0–0), 11	0.38 (1.2)	0.7 (2.5)	−0.3 (−1.7 to 0.7)	.396
CRA	0		0	0	0	
	*N* (%)		*n* (%)	*n* (%)	Difference %- points	
Number of patients admitted after initial stay	38 (93%)		21 (100%)	17 (85%)	15	.069
MHH	21 (51%)		9 (43%)	12 (60%)	− 17	
CMHC	17 (41%)		4 (19%)	13 (65%)	− 46	
CRA aftercare	22 (54%)		21 (100%)	1 (5%)	95	
CRA self-referral	8 (20%)		7 (33%)	1 (5%)	28	
Number of patient with readmission	15 (37%)		6 (29%)	9 (45%)	− 16	.281
MHH	12 (29%)		6 (29%)	6 (30%)	−1	
CMHC	6 (15%)		2 (10%)	4 (20%)	−10	
CRA	0		0	0	0	

MHH Mental Health hospital, CMHC Community mental health centre, CRA Community residential aftercare

^a^One patient in the control group, who after the initial stay at the MHH was discharged to home, later had several acute admissions to the MHH. After the last of these, the patient was discharged to the CRA from the MHH as the CMHC declined the referral of this patient from the MHH, and a solution had to be found. This patient then stayed at the CRA for 255 days

^b^One patient in the control group was admitted to a self-referral bed at the CRA from the patient’s residence by a community mental health team as an emergency measure due to lack of other suitable services. The patient stayed at the CRA for 12 days

The number of and proportion of persons with admissions and readmissions was not statistically significant different between the groups, but was slightly lower in the intervention group (Table 5).

The total cost for all mental health services for 12 months was 38.5% lower for patients randomised to the CRA (mean differences − 23,071 EUR, 95% CI -45,450 to 3027, *p* = .057) (Table 6). This was mainly due to lower inpatients costs which had a mean difference of − 17,741 EUR (95% CI -36,824 to 4503, *p* = .042) in favour of the intervention.

**Table 6:** Cost for all inpatient and outpatient services from baseline (date of randomisation) to twelve months. Values are in euro (EUR)

Variable	All (n = 41)		Intervention (N = 21)	Control (N = 20)	Between groups	P- value
	Mean (SD)	Median (IQR), range	Mean (SD)	Mean (SD)	Mean diff (95% CI)	
Total cost	48,131 (39726)	31,232 (20813–57,209), 149,638	36,877 (32647)	59,948 (43745)	−23,071 (−45,450 to 3027)	.057
Sum inpatient service cost	38,321 (34137)	25,825 (14808–46,641), 142,643	29,667 (31543)	47,408 (35161)	−17,741 (−36,824 to 4503)	.042
MHH	18,920 (26663)	8518 (3194–19,167), 103,821	11,941 (21085)	26,248 (30304)	−14,306 (−31,754 to 912)	.008
CMHC	11,073 (19526)	8518 (3194–19,167), 74,237	4896 (13812)	17,559 (22703)	−12,663 (−23,568 to − 2632)	.004
CRA	8018 (13573)	539 (0–12,810), 68,770	12,380 (10142)	3428 (15377)	8941 (− 608 to 16,194)	.000
CRA – self-referral	309 (749)	0 (0–0), 3236	449 (764)	161 (723)	287 (− 199 to 714)	.035
Sum outpatient services cost	9809 (12934)	4916 (875–15,192), 49,443	7210 (9578)	12,539 (15501)	− 5329 (−13,216 to 2546)	.215
Mental health outpatient treatment	2678 (7128)	486 (0–2178), 43,757	2145 (3603)	3239 (9625)	− 1094 (− 6125 to 2240)	.843
Mental health ambulant treatment	1786 (4050)	0 (0–180), 15,619	620 (1920)	3010 (5250)	− 2389.7 (− 4681 to 90.6)	.160
Home help (cleaning, shopping etc.)	153 (558)	0 (0–0), 3313	116 (310)	191 (743)	−74.5 (− 412.6 to 214.5)	.680
Home care nursing	4661 (8966)	0 (0–4165), 33,460	3999 (9359)	5357 (8721)	−1357.7 (− 6520 to 4051)	.023
Community mental health consultation	530 (1864)	0 (0–300), 11,605	328 (742)	741 (2577)	−413.6 (− 1773 to 411)	.758

### Post hoc analysis

The post hoc analysis was done due to the observed differences in patient characteristics at baseline, using a multivariable linear regression model with total cost as the independent variable and the four baseline characteristics with the largest %-point differences between the groups (> 20%-points, Table 2) as dependent variables. The difference between the groups in favour of the intervention group was a reduced cost of − 19,781 EUR (95% CI -44,072 to 4509, *p*=,107) in the best model, which included three of the four independent variables (Homeless, Diagnosis and Employment status).

## Discussion

This is the first RCT study on the effect of discharge for patients with SMI to a community residential aftercare facility (CRA) with no organised in-house activities or on-site treatment. The differences in utilisation and cost during 12 months were in favour of the intervention group, but mostly with *p*-values above the conventional cut-off *p* < 0.05. The confidence intervals were wide, meaning that there was imprecision in the estimates. Thus, no final conclusion on the effect of the CRA can be made based on this study.

However, the study gives indication of a potential effect of discharging patient in need of community aftercare to the CRA. The best estimates for this potential based on the present study is that it can reduce the use of hourly based community mental health services with 29% (22 h), with a cost saving of 29% (1845 EUR) for each patient compared to usual care. The total number of inpatient days for one year was the same (66 days), but the number of inpatient days in the mental health hospital was 54% (13 days) lower. Importantly, although using less services, the point estimate for the number of inpatient admissions and readmissions was respectively 18% (− 0.9 admissions) and 42% (− 0.8 readmissions) lower in the intervention group indicating at least no major worsening in the intervention group.

Considering possible mechanisms and explanations for the direction of the observed effect, it seems that the CRA is successful in facilitating independent living which, in turn, leads to less mental health service use. Even if a stay at the CRA does not reduce the total number of inpatient days during the first year, spending more time in residential aftercare service can leave room for better assessment of and subsequent alignment between the patients’ actual care needs in the community and the services offered. Another explanation can be that when the hospital staff communicate the care needs of the patient to the community services, they do so based on what they have seen during the hospital stay (observer bias) [30]. This can differ from the patients’ behaviour in a CRA setting where there are no in-house organised activities, and where, consequently, the staff can observe how the patient manages in a more home like setting. In addition, a stay in the CRA allows for more time in assessing and setting up the required level of services to support independent living. This is in line with the finding in an observational cohort study among six community residential alternatives compared to six standard acute wards [18], which found that patients having used the community alternative had more contact with community mental health teams, early intervention services and crisis teams.

Furthermore, the patient’s own role can be important, especially what the patients can learn and do differently after a stay at a “boring hotel” [26]. This study cannot answer whether the CRA might increase the patients’ contribution or abilities such as agency, responsibility, self-management, coping and empowerment. One way of looking at independence is by examining motivation and behaviour, as the CRA attempt to motivate the patients to adopt a more independent behaviour. The self-determination theory (SDT) can shed some light on this, as it claims to provide a universal framework for understanding the individual and environmental factors that shape motivation and subsequent behaviour [31]. According to SDT, motivation depends on the (lack of) support for three basic psychological needs: autonomy, competence, and relatedness. It does not seem to be fare fetched to suggest that these areas were strengthened; leading to the patients’ feeling more equipped to do self-care activities such as preparing their own meals, structuring their daily routines and introducing activities in their neighbourhood.

The chosen primary outcome, use of hourly based community health and social services, was chosen based on an assumption that discharge to the CRA would help identify the best level of service for each patient, which was expected to be lower than usual care. This does not imply that less use of community health and social services was a desired outcome by itself. The aim must be to balance the level of services to the patient’s needs. However, with the aim to promote independency among service users, the level of services should not be so high as to jeopardise this. To be almost self-reliant and be in command of one’s own life are basic rights that most humans takes for granted. Given the direction of the results in this study, pointing towards both less use of services and fewer re−/admissions for those randomised to the CRA, there are indications that having a strong community orientation in the discharge process can result in a service level promoting independency.

Even if both step-up [15–19] and step down [20–23] community residential services exist, none of the studies investigating the effects and costs are directly comparable to this study, as they offer in-house activities or treatment. However, according to these studies, there seems to be a clear indication that community residential services can reduce costs [17, 18, 20], similar to the point estimates found in this study of around 1/3 reduction: Byford et al. [17] found 22% lower total 12-month costs (£14,952 vs. £19,288), a UK based study by Slade et al. [18] reported 61% lower 12-month inpatient costs (£3832 vs. £9850) and Thomas et al. in Australia [20] found that the cost per day per client in the step-up step-down program was 32% lower ($517 vs. $758). The explanation for reduced costs in these studies and in our study, is chiefly due to reduced inpatient stays and use of specialist services.

We did not measure change in patients’ level of symptoms and functioning, but two other studies on community residential aftercare have done this [21, 23]. An observational study from Australia [21] found improvement in patients’ symptoms and functioning three months after discharge from the residential inpatient step-down unit. An RCT among justice involved persons [23] found a significant reduction in psychiatric symptom severity after two years in those who had been admitted to self-run community residential aftercare (Oxford House).

### Strength and limitations

To the best of our knowledge, this is the first RCT-study to investigate a step-down model of a staffed residential aftercare not offering in-house activities or treatment therapy. The strength of this study is the use of data from health service registers covering both specialist and community mental health care utilisation, which provided complete data on all participants.

One major limitation was that the sample size was smaller than what was pre-planned, which in addition to giving imprecise estimates, also is the most likely explanation for the differences in patient characteristics at baseline. An alternative explanation of baseline difference is flaws in the randomisation and allocation process. However, the randomisation was internet based and it was not possible for anyone involved in the study processes to influence the allocation.

The recruitment was both slow and low despite a range of study activities from information meetings to encouragement from management. The main reason expressed by some of the inpatient staff in the MHH was scepticism about the level of competence at the CRA, particularly the lack of psychologists. This scepticism was surprising as the CRA had been in operation before the study and should thus be known to the hospital staff with treatment responsibility. Furthermore, it cannot be ruled out that the persons with treatment responsibility recruiting patients to the CRA previously and maybe to the study represent a sub-set, as it was not collected data on who recruited patients.

Another reason for the recruitment problem can be that the staff at the hospital did not include patients for this study to avoid them being randomised to the control group, which meant that they would get a delayed discharge compared to being discharged to the CRA. This suspicion is strengthening by the fact that some patients were discharged directly to the CRA instead being recruited to the study.

Nevertheless, it is a major limitation that the number of eligible patients was not recorded. However, it is obvious that the number of eligible patients was far higher than the 41 patients recruited, as the recruitment took place in an 81-bed unit over 26 months. Thus, caution is needed when generalising the result of this study to other settings, as the sample in this study represents a subset of hospitalised persons with SMI. The best description of this subset is that it is representative of the group of patients with SMI that were considered suitable for the CRA by personnel with treatment responsibility in the hospital who are willing to refer patients to the CRA. This assumption is strengthened by the contact nurses for the study who reported that almost all participants that were introduced to the study agreed to participate.

## Conclusion

In this study, it was not possible to draw a definite conclusion about the effect, due to the small sample and imprecision of the estimates. The direction of the results and size of the point estimate, including findings in other studies, indicates that transferring patients ready for discharge from mental hospital to community residential aftercare without organized in-house activities has the potential to reduce total consumption of health services and costs without increased hospital admissions.

## Abbreviations

CMHC: Community mental health centre
CRA: Community residential aftercare
MHH: Mental health hospital
RCT: Randomized controlled trial
SMI: Severe mental illness
